# Priming of SARS-CoV-2 S protein by several membrane-bound serine proteinases could explain enhanced viral infectivity and systemic COVID-19 infection

**DOI:** 10.1074/jbc.REV120.015980

**Published:** 2020-12-06

**Authors:** Pablo Fuentes-Prior

**Affiliations:** Molecular Bases of Disease, Biomedical Research Institute (IIB) Sant Pau, Hospital de la Santa Creu i Sant Pau, Barcelona, Spain

**Keywords:** structure–function relationship, TMPRSS2, membrane-associated serine proteinases (MASPs), serpins, HAI-1/SPINT1, coronaviruses, COVID-19, cell tropism, spike (S) protein, viral fusion, α2-AP, α2-antiplasmin, ACE2, angiotensin-converting enzyme 2, MASPs, membrane-associated serine proteinases, MERS, Middle East respiratory syndrome, PAI-1, plasminogen activator inhibitor 1, PCI, protein C inhibitor, QM/MD, quantum mechanical/molecular dynamics, SARS, severe acute respiratory syndrome, uPA, urokinase-type plasminogen activator

## Abstract

The ongoing COVID-19 pandemic has already caused over a million deaths worldwide, and this death toll will be much higher before effective treatments and vaccines are available. The causative agent of the disease, the coronavirus SARS-CoV-2, shows important similarities with the previously emerged SARS-CoV-1, but also striking differences. First, SARS-CoV-2 possesses a significantly higher transmission rate and infectivity than SARS-CoV-1 and has infected in a few months over 60 million people. Moreover, COVID-19 has a systemic character, as in addition to the lungs, it also affects the heart, liver, and kidneys among other organs of the patients and causes frequent thrombotic and neurological complications. In fact, the term “viral sepsis” has been recently coined to describe the clinical observations. Here I review current structure–function information on the viral spike proteins and the membrane fusion process to provide plausible explanations for these observations. I hypothesize that several membrane-associated serine proteinases (MASPs), in synergy with or in place of TMPRSS2, contribute to activate the SARS-CoV-2 spike protein. Relative concentrations of the attachment receptor, ACE2, MASPs, their endogenous inhibitors (the Kunitz-type transmembrane inhibitors, HAI-1/SPINT1 and HAI-2/SPINT2, as well as major circulating serpins) would determine the infection rate of host cells. The exclusive or predominant expression of major MASPs in specific human organs suggests a direct role of these proteinases in *e.g.*, heart infection and myocardial injury, liver dysfunction, kidney damage, as well as neurological complications. Thorough consideration of these factors could have a positive impact on the control of the current COVID-19 pandemic.

Coronaviruses cause important diseases in humans, most notably severe acute respiratory syndrome (SARS) and Middle East respiratory syndrome (MERS). According to the currently accepted mechanism of viral infection, both SARS-CoV (also termed SARS-CoV-1) and the related, recently emerged SARS-CoV-2 rely on two membrane-bound host peptidases for entry into target cells: the carboxypeptidase, angiotensin-converting enzyme 2 (ACE2), and a serine proteinase known as TMPRSS2/TMPS2 or epitheliasin. (TMPRSS stands for “Transmembrane Protease, Serine”). The protruding spike (S) glycoproteins of SARS-CoVs interact first with ACE2 and are then proteolytically cleaved by TMPRSS2 to trigger fusion of the viral envelope with the host cell membrane (1, 2, 3, 4, 5, 6, 7, 8, 9). (See Fig. 1*A* for the domain organization of the viral S protein and Fig. 1, *B*–*C* for its three-dimensional (3D) structure). TMPRSS2 plays a dual role in the infection process as it also cleaves ACE2, which increases uptake of SARS-CoV and likely also SARS-CoV-2 virions (3). In addition, TMPRSS2 also activates other human pathogenic coronaviruses that cause the common cold as well as several strains of influenza A viruses, although these pathogens use unrelated attachment receptors for host cell entry (10).

**Figure 1:**
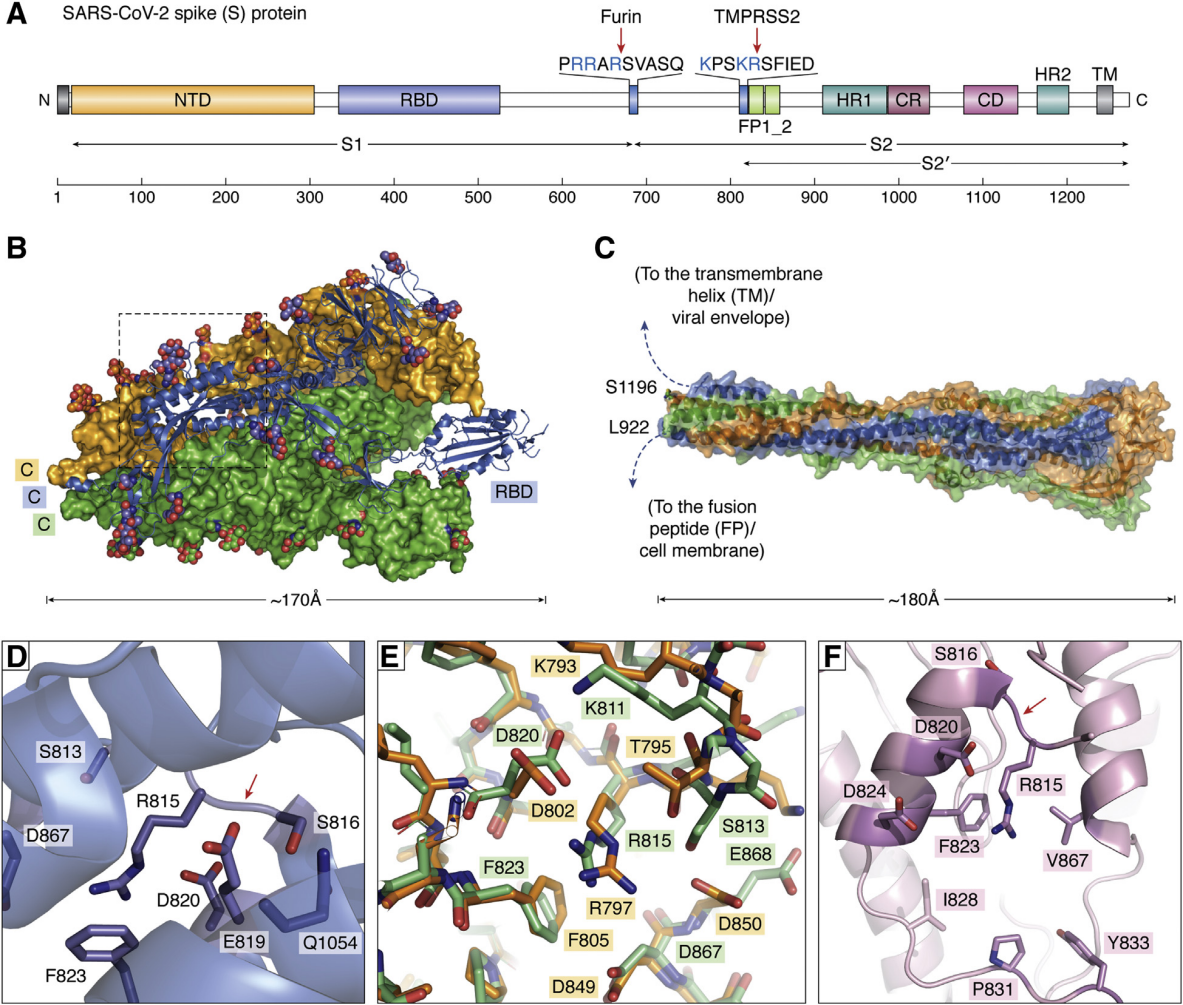
**The SARS-CoV-2 spike (S) protein is responsible for viral attachment to host cells and membrane fusion.***A*, schematic representation of domain organization. *B*, 3D structure of trimeric S glycoprotein in the prefusion conformation (after PDB 6VSB). The RBD of one monomer is in the “up” conformation, *i.e.*, exposed for interaction with the human ACE2 ectodomain (*blue*), while the two other monomers are depicted as *green* and *light-orange* solid surfaces. Note that carbohydrate chains (*color-coded spheres*) are distributed all over the surface of the trimer. *C*, postfusion conformation of the viral S protein. After priming, the heptad repeats and central helix from each S2’ monomer adopt extended, tightly packed continuous α-helical structures that generate a 180-Å-long, cone-shaped arrangement with a prominent triple-helical core. (Here, the structure of the highly related murine hepatitis virus is represented; PDB 6B3O, ref. (161)). Residues topologically equivalent to Leu922 and Ser1196 of each monomer are located less than 20 Å apart. In this manner, the rearranged S trimer brings viral and host cell membranes into close proximity to allow their fusion and infection. *D*, S2’ cleavage site (Arg815-Ser816, *red arrow*), highlighting the solvent-protected conformation of these residues, which is incompatible with proteinase binding and proteolysis: the Arg815 side chain is clamped by acidic residues (Asp820, Asp867) and contacts with Phe823, while the side chain of Ser816 points inward and is fixed by those of Glu819 and Gln1054. *E*, comparison of S2’ priming sites in SARS-CoV-1 and 2. Several critical residues around the Arg815 side chain (Arg797 in SARS-CoV-1) are color-coded (carbon atoms are *orange* and *green* in SARS-CoV-1 and 2, respectively). Different conformations for most of the activation loop (*upper right part of the panel*) leads to significant changes in the shielding of the Arg815 side chain; Ser813→Thr797 and Glu868→Asp850 replacements in particular generate a “cage” better suited to hold the Arg815 side chain in its protected conformation. *F*, structure of the Arg815-binding pocket in MERS-CoV S protein (after PDB 5X59). As a major difference with SARS-CoV-1 and 2, the connector topologically equivalent to Val826-Gln836 in SARS-CoV-2 is defined and forms a lid that would sterically clash with an approaching proteinase. This lid projects the side chains of Ile828, Pro831 and Tyr833 toward Arg815, which, together with the replacement of Asp867 by an aliphatic valine, makes the Arg815 cage much more apolar in MERS-CoV. Conceivably, these subtle sequence and structure variations facilitate exposure and cleavage of the Arg815-Ser816 peptide bond SARS-CoV-2 and thus contribute to its increase infectivity compared with other related coronaviruses. (To facilitate comparisons, residues are numbered according to their topological equivalences in SARS-CoV-2).

The extraordinarily rapid spread of the COVID-19 pandemic caused by SARS-CoV-2, the absence of a vaccine and of pharmacological drugs capable of inhibiting viral infection and its large economic impact have spurred intense research on the cell entry mechanism, a key factor in viral pathogenesis. Among several unresolved issues, there are two questions of particular clinical relevance. First, why does the virus show a much higher transmission rate and infectivity than SARS-CoV, despite highly similar protein structures, including about 90% conserved residues of their spike proteins? Second, why is SARS-CoV-2 capable of infecting different human organs, in contrast to the rather restricted cell tropism of SARS-CoV? A plausible answer to these questions follows, based on a careful analysis of available structure–function information regarding coronavirus spike proteins and the membrane fusion process.

## Furin cleavage preactivates SARS-CoV-2 spike proteins

The spike proteins of SARS-CoV-1 and 2 are activated (“primed”) upon proteolysis of two peptide bonds: the first one, corresponding to Arg685-Ser686 in the SARS-CoV-2 protein, separates the so-called S1 and S2 subunits (Fig. 1*A*) (2). The N-terminal S1 subunit contains the receptor-binding domain (RBD) responsible for ACE2 binding (8, 11, 12), while the C-terminal S2 subunit remains attached to the viral envelope after proteolysis at the S1/S2 site and is ultimately responsible for its fusion with the host cell membrane. This initial cleavage is followed by proteolysis at the S2’ site (Arg815-Ser816 in SARS-CoV-2; Fig. 1*D*), which exposes the highly antigenic Ser816-Phe833 fusion peptide, and elicits major, irreversible conformational changes. This results in a radically different conformation of the S protein, which is competent for membrane fusion and infection (see *e.g.*, refs. (2, 13) and Fig. 1*C*).

As a major difference with SARS-CoV, the S1/S2 peptide bond in SARS-CoV-2 S proteins is efficiently severed by another host cell proteinase, the proprotein convertase furin, within the secretory pathway of infected cells (7). (For a review on the roles of furin in homeostasis and disease, see ref. (14)). Because of furin-mediated proteolysis, the spike proteins in the SARS-CoV-2 virions are found in a preactivated state and require cleavage of a single bond, Arg815-Ser816, for activation of the fusion machinery. This unique feature of SARS-CoV-2 is essential for S protein-mediated cell–cell fusion and entry into human cells and thus, an important determinant of viral infectivity and pathogenesis (6).

## Several membrane-associated serine proteinases might synergize with or replace TMPRSS2 as cellular activator of SARS-CoV-2

**Figure 2:**
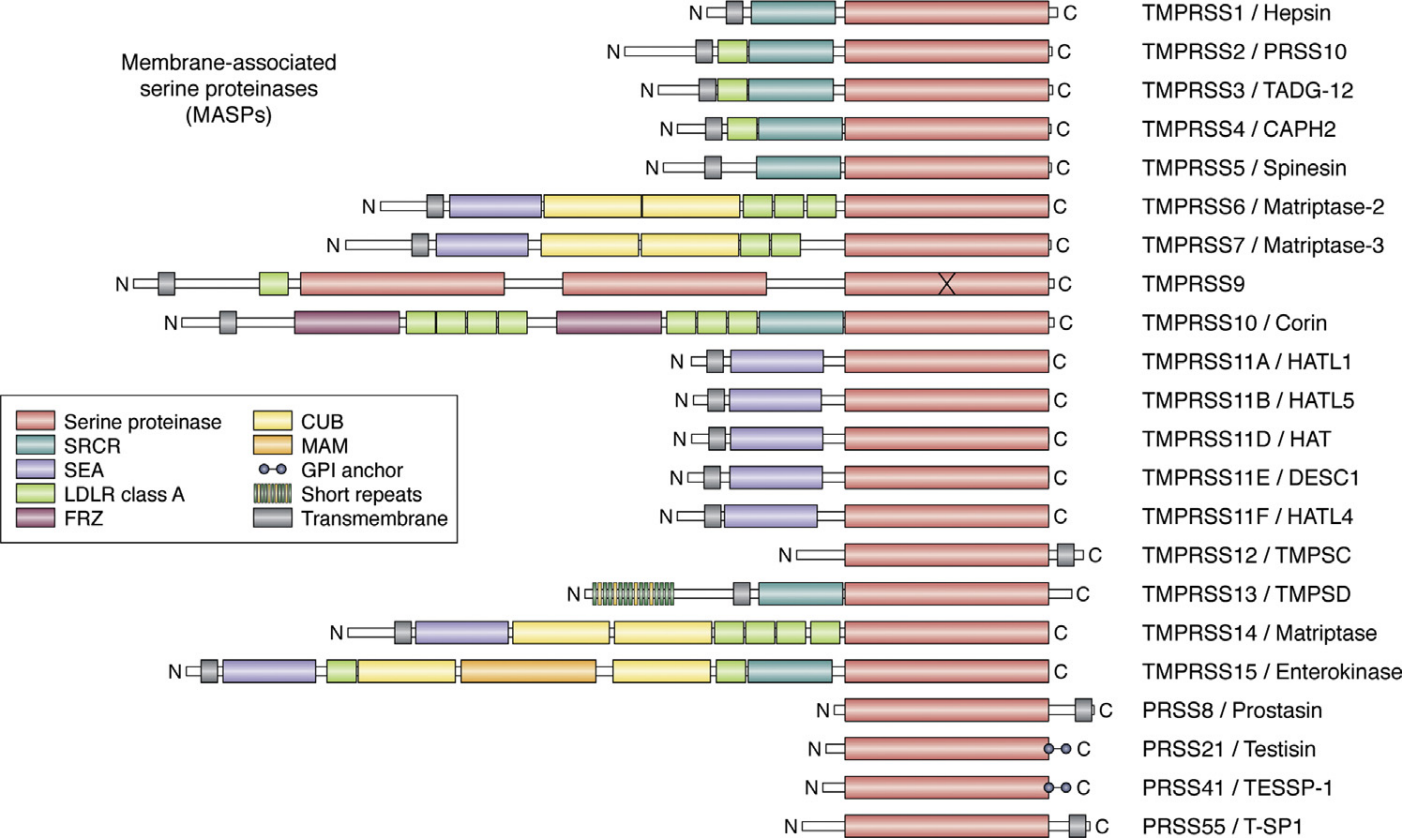
**Membrane-associated serine proteinases (MASPs): mosaic proteinases for cleavage of transmembrane and membrane-proximal substrates.** Structural domains (approximate scale) are color-coded. Note the variety of noncatalytic scaffolds found in these mosaic proteinases, most notably in the matriptases (TMPRSS6, -7 and -14), corin/TMPRSS10, and enterokinase/TMPRSS15. In addition to type II transmembrane serine proteinases (TTSPs), type I or glycosylphosphatidylinositol (GPI)-anchored proteins that possess similar catalytic domains are also included (TMPRSS12/TMPSD, PRSS8/prostasin, PRSS21/testisin, PRS41/TESSP-1, and PRS55/T-SP1). CUB, complement C1r/C1s, Uegf, Bmp1; FRZ, frizzled; LDLR, low-density lipoprotein receptor; MAM, meprin, A-5 protein, and receptor protein-tyrosine phosphatase mu; SEA, sea urchin sperm protein, enterokinase, agrin; SRCR, scavenger receptor cysteine-rich.

**Figure 3:**
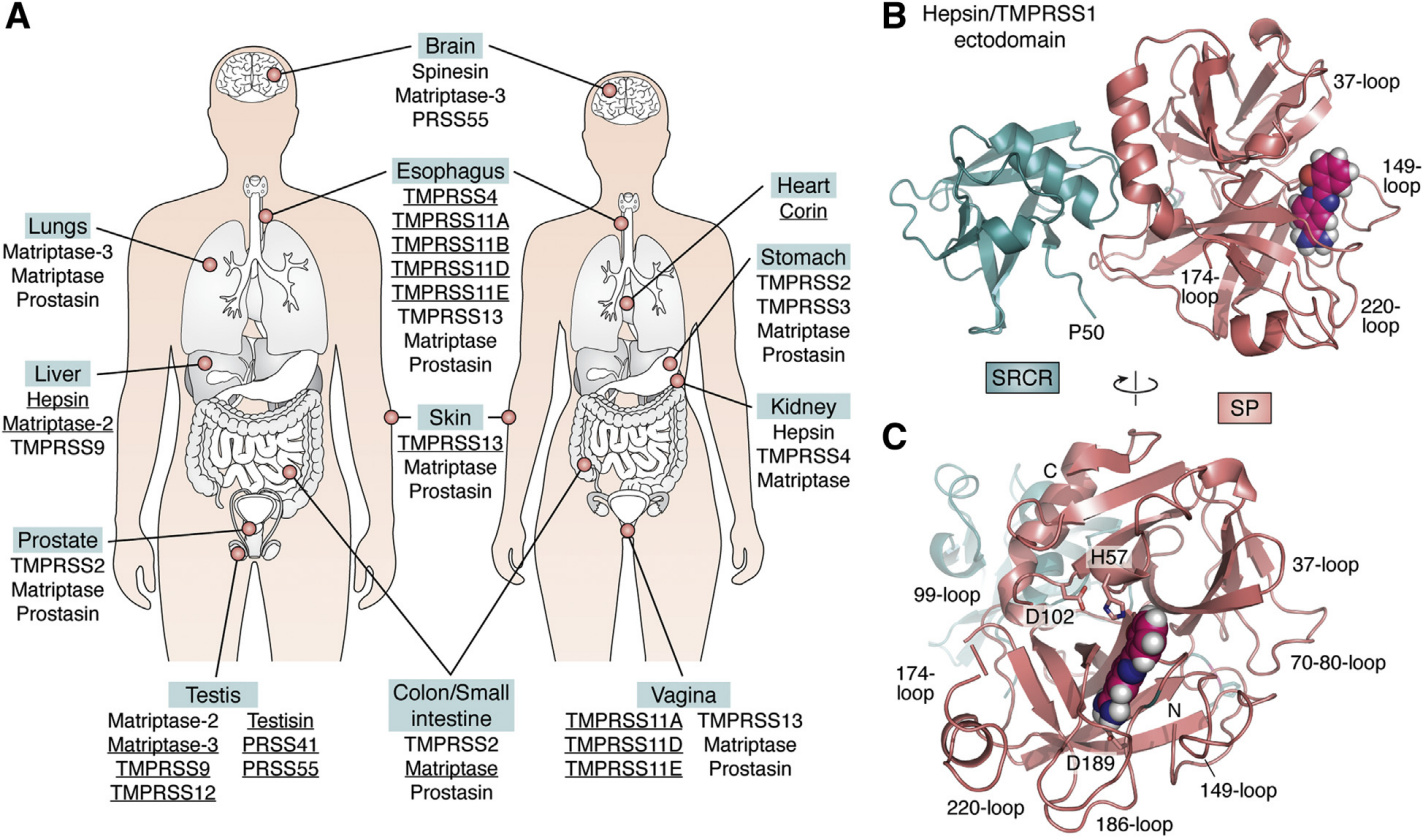
**Expression patterns and structure of membrane-associated serine proteinases.***A*, organs with highest expression levels of specific MASPs. Data for some organs are indicated only for males or females, although there are no known sex-associated differences in MASP expression in these organs. Data from the GTEx Portal (gtexportal.org) (see also Fig. 4*A*). *B*–*C*, 3D crystal structure of human hepsin/TMPRSS1 ectodomain, represented as a cartoon highlighting major secondary structure elements; loops that shape the active-site cleft are noted. (After PDB entry 1P57: N-terminal SRCR module (*deep-teal cyan*); serine proteinase domain (*deep salmon-red*)). A small-molecule inhibitor bound in the S_1_ specificity pocket of the serine proteinase domain (2-{5-[amino(iminio)methyl]-1H-benzimidazol-2-YL}benzenolate) is shown as color-coded spheres (carbon, *pink*; oxygen, *red*; nitrogen, *blue*; and hydrogen, *gray*). *B*, side view, highlighting the proximity of the N-terminal residue of the SRCR domain, Pro50, to the transmembrane helix, Gly24-Leu44. This locates also the rigidly attached catalytic domain of the proteinase essentially flat against the cell membrane (74). A similar localization should be expected for the catalytic domains of other MASPs, which implies that the Arg815-Ser816 bond in the S protein would be cleaved close to the cell membrane, facilitating rapid interaction with the exposed viral fusion peptide and escape from immune surveillance. *C*, view of the proteinase in the “standard orientation”, *e.g.*, with active-site residues (given with all their nonhydrogen atoms, color-coded) facing the viewer and substrates running from left to right. The N and C termini of the catalytic chain are noted (Ile16 and Thr253, respectively) as well as Asp189 at the bottom of the S_1_ pocket, which is largely responsible for the recognition and cleavage of substrates after a basic Arg/Lys residue.

**Figure 4:**
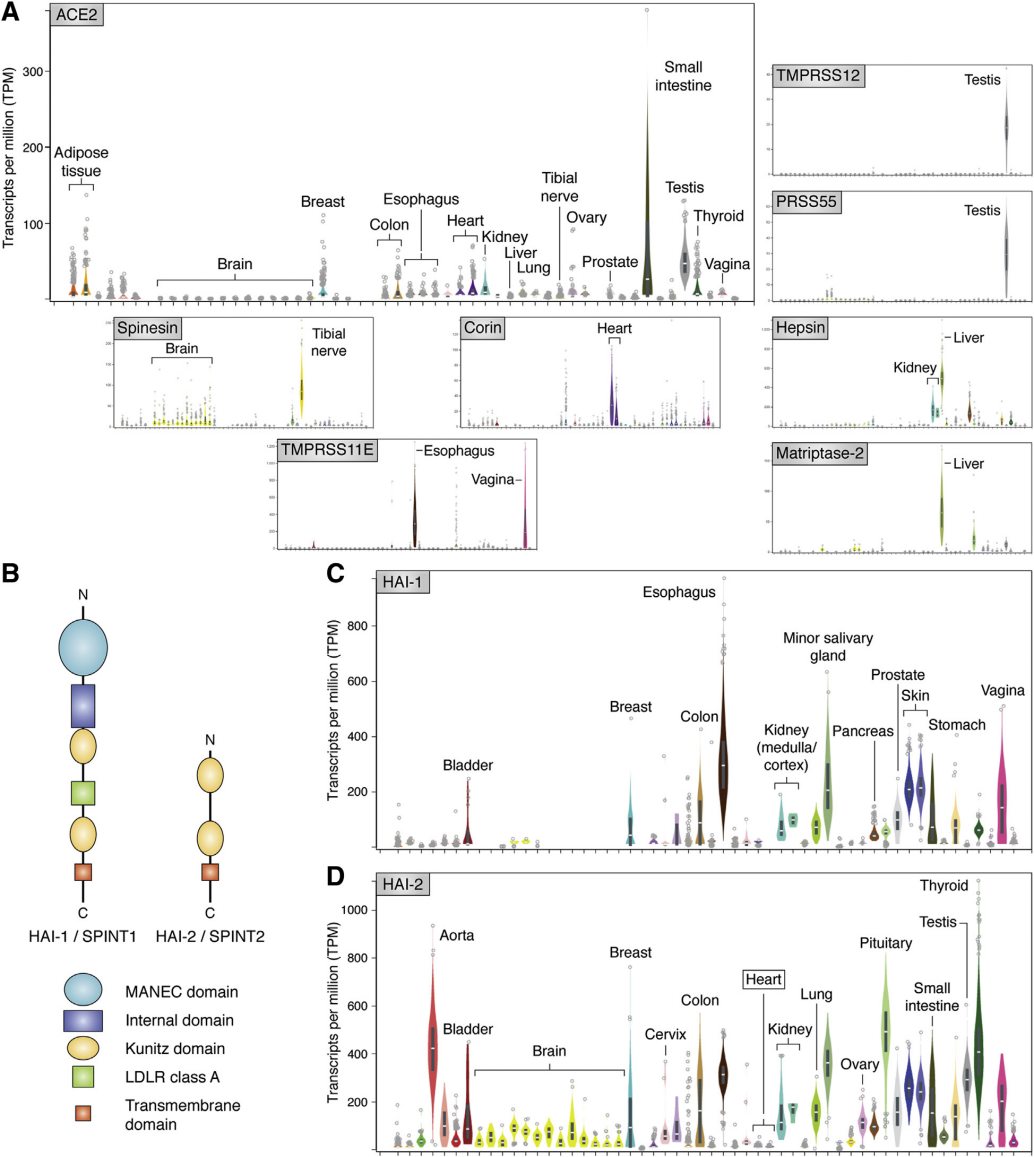
**Expression patterns of human ACE2, MASPs and their endogenous inhibitors.***A*, comparison of the expression levels of human ACE2 and a selection of those MASPs with restricted expression patterns. Spinesin/TMPRSS5 is predominantly expressed in the brain and the tibial nerve, corin in the heart, and TMPRSS12, PRSS21, PRSS41 as well as PRSS51 in the testis. Further, matriptase-2 and hepsin are expressed at highest levels in the liver or in liver and kidney, respectively. The members of the airway tract subgroup, TMPRSS11A–11F are predominantly expressed in the mucosa of the esophagus and in the vagina. *B*, schematic representation of domain organization in the endogenous Kunitz-type inhibitors of membrane-associated serine proteinases, HAI-1/SPINT1 and HAI-2/SPINT2. *C*–*D*, expression patterns of human HAI-1 (*C*) and HAI-2 (*D*). Note the overall complementarity of expression profiles in different organs (*e.g.*, HAI-2 but not its paralog is expressed in the brain and arteries), although both inhibitors are similarly expressed in several organs, including kidneys, prostate, and vagina. The extremely low expression of both Kunitz inhibitors in the heart is also noteworthy, which suggests that the proteolytic activity of the heart-specific MASP, corin/TMPRSS10, would only be controlled by circulating serpins. The expression values are given as Transcripts per Million (TPM), as reported in the GTex Portal (gtexportal.org). HAI, hepatocyte growth factor activator inhibitor; MANEC, motif at N-terminus with eight-cysteines; SPINT, serine protease inhibitor Kunitz type.

The canonical activator of SARS-CoV-1/-2, TMPRSS2, belongs to a subfamily of multidomain, transmembrane serine proteinases with important roles in development and homeostasis (15, 16, 17, 18, 19). (The domain organization of TMPRSS2 and related, membrane-associated serine proteinases (MASPs) is schematically represented in Fig. 2; relevant information on these enzymes is summarized in Table 1). Notably, most MASPs are exclusively or predominantly expressed in specific human organs, including the heart (corin/TMPRSS10), liver (hepsin/TMPRSS1 and matriptase-2/TMPRSS6), brain (spinesin/TMPRSS5), esophagus (TMPRSS4 and TMPRSS11A to F), prostate (TMPRSS2 and prostasin/PRSS8), and testes (matriptase-3/TMPRSS7, TMPRSS12, testisin/PRSS21, TESSP-1/PRSS41, and T-SP1/PRSS55) (Figs. 3*A* and 4*A*).

**Table 1:** Summary of information regarding human membrane-associated serine proteinases (MASPs)

Recommended MASP name^a^/Other names	EC/MEROPS^b^	Gene name	Length (residues)	Expression pattern and relevance for pathology
Hepsin TMPRSS1	3.4.21.106/S01.224	*HPN* (*TMPRSS1*)	417	Expressed at highest levels in the liver. Also expressed at high levels in the kidneys and pancreas. Other expression organs: stomach and thyroid gland. Overexpressed in several primary tumors, in particular in prostate and ovarian cancers.
TMPS2 TMPRSS2, epitheliasin, serine protease 10 (PRSS10)	3.4.21.-/S01.247	*TMPRSS2* (*PRSS10*)	492	Highest expression in the prostate, stomach, small intestine, and colon. Expressed also in the airway and alveolar cells. Overexpressed in most prostate cancer patients. Proteolytically cleaves and activates SARS-CoV-1 and 2 as well as MERS-CoV S proteins, which triggers virus–cell membrane fusion.
TMPS3 TMPRSS3, tumor-associated differentially expressed gene 12 protein (TADG-12)	3.4.21.-/S01.079	*TMPRSS3* (*ECHOS1*, *TADG12*)	454	TTSP also reported as an endothelium reticulum protein. Expressed in the inner ear and linked to deafness. Expressed also at low levels in the stomach and breast, among other organs.
TMPS4 TMPRSS4, channel-activating protease 2 (CAPH2), membrane-type serine protease 2 (MT-SP2)	3.4.21.-/S01.034	*TMPRSS4*	437	Highest expression levels in the esophagus. Broadly expressed in the kidney, bladder, colon, small intestine, prostate, and vagina, among other tissues. Upregulated in prostate, colon, and gastric cancers.
TMPS5 TMPRSS5, spinal cord–enriched trypsin-like protease (spinesin)	3.4.21.-/S01.313	*TMPRSS5*	457	Predominantly expressed in neurons, their axons, and at the synapses of motor neurons in the spinal cord. Expressed at highest levels in the tibial nerve. Expressed throughout the brain.
TMPS6 TMPRSS6, matriptase-2 (MT2)	3.4.21.-/S01.308	*TMPRSS6*	811	Highest expression levels in the liver. Also expressed in the testis and in the pituitary gland. Plays a critical role in the regulation of iron homeostasis through cleavage of the cell surface BMP coreceptor, hemojuvelin.
TMPS7 TMPRSS7, matriptase-3 (MT3)	3.4.21.-/S01.072	*TMPRSS7*	843	Highest expression levels in the testis. Expressed also at significant levels in the skin, brain, lungs, as well as pituitary, thyroid, and minor salivary glands.
TMPS9 TMPRSS9, polyserine protease 1, polyserase-1	3.4.21.-/S01.357	*TMPRSS9*	1059	Highest expression levels in the testis, but also expressed in the spleen and liver.
Corin TMPRSS10, atrial natriuretic peptide-converting enzyme, heart-specific serine proteinase ATC2	3.4.21.-/S01.019	*CORIN* (*CRN*, *TMPRSS10*)	1042	Highly expressed in the heart (left ventricle and atrial appendage). Also expressed in the skin, uterus, and vagina, among other organs. Activates the cardiac hormone, atrial natriuretic peptide, and thus involved in blood pressure regulation.
TM11A TMPRSS11A, airway trypsin-like protease 1, (HATL1), esophageal cancer-susceptibility gene 1 protein, epidermal type-II transmembrane serine protease	3.4.21.-/S01.292	*TMPRSS11A* (*HATL1*, *ECRG1*, *HESP*)	421	Expressed at highest levels in the esophagus, vagina, and cervix. Also expressed in the lungs.
TM11B TMPRSS11B, airway trypsin-like protease 5 (HATL5)	3.4.21.-/S01.365	*TMPRSS11B*	416	Expressed at highest levels in the cervix, esophagus, and oral cavity. Significantly decreased in cervical, esophageal, and head and neck carcinomas. Highly upregulated in the lung squamous cell carcinoma.
TM11D TMPRSS11D, airway trypsin-like protease (HAT)	3.4.21.-/S01.047	*TMPRSS11D* (*HAT*)	418	Expressed at highest levels in the vagina and esophagus. Expressed also in the cells of the submucosal serous glands of the bronchi and trachea.
TM11E TMPRSS11E, differentially-expressed in squamous cell carcinoma gene 1 (DESC1)	3.4.21.-/S01.021	*TMPRSS11E* (*DESC1*)	423	Expressed at highest levels in the esophagus and vagina. Also expressed in the bladder. Upregulated in tumors of different origin.
TM11 F TMPRSS11 F, airway trypsin-like protease 4 (HATL4)	3.4.21.-/S01.321	*TMPRSS11F* (*HATL4*)	438	Expressed at highest levels in the esophagus and vagina. Also expressed in the skin. Unique function in epidermal barrier formation.
TMPSC TMPRSS12	3.4.21.-/S01.291	*TMPRSS12*	348	Type I transmembrane protein expressed almost exclusively in the testis. Expressed in colorectal cancer.
TMPSD TMPRSS13, membrane-type mosaic serine protease (MSP, MSPL, MSPS)	3.4.21.-/S01.087	*TMPRSS13* (*MSP*, *TMPRSS11*)	586	Predominantly expressed in the skin. Also expressed in the esophagus and vagina, among other organs. Cell-surface expression regulated by phosphorylation of its intracellular peptide.
ST14 TMPRSS14, matriptase, membrane-type serine protease 1 (MT-SP1), tumor-associated differentially expressed gene 15 protein (TADG-15), epithin, SNC19	3.4.21.109/S01.302	*ST14* (*PRSS14*, *SNC19*, *TADG15*)	855	Ubiquitously expressed, at highest levels in the colon and small intestine, but also in the skin, lung, kidneys, breast, and vagina. Proteolytic activity essential for epithelial integrity. Implicated in the development and progression of several cancers. Proposed to play a role in breast cancer invasion and metastasis. Forms reciprocal zymogen activation complex with prostasin.
Enteropeptidase TMPRSS15, enterokinase, serine protease 7 (PRSS7)	3.4.21.9/S01.156	*TMPRSS15* (*ENTK*, *PRSS7*)	1019	Expression restricted to the small intestine. Starts a zymogen cascade leading to digestive enzyme activation by converting trypsinogen to trypsin.
Prostasin PRSS8, channel-activating protease-1 (CAP1), serine protease 8 (PRSS8)	3.4.21.-/S01.159	*PRSS8*	343^c^	Type I transmembrane protein with a preference for polybasic substrates.^d^ Ubiquitously expressed, at highest levels in the colon and small intestine, but also in the liver, salivary gland, kidney (renal proximal tubular cells), lung, skin. Forms reciprocal zymogen activation complex with matriptase.
Testisin PRSS21, eosinophil serine protease 1 (ESP1), TESP5	3.4.21.-/S01.011	*PRSS21* (*ESP1*, *TEST1*)	314^e^	GPI-anchored protein. Expressed only in the testis but lost in testicular tumors. Also expressed in the minor salivary gland and lungs. Important for sperm cell maturation and fertilizing ability.
PRSS41 Testis serine protease 1 (TESSP-1)	3.4.21.-/S01.417	*TESSP1*	318^f^	Testis-specific GPI-anchored protein. Also expressed in the minor salivary gland and lungs. Required for the progression of meiosis during spermatogenesis.
PRSS55 Testis serine protease 1 (T-SP1)	3.4.21.-/S01.299	*PRSS55* (*TSP1*)	352^g^	Type I transmembrane protein.^h^ Expressed at highest levels in the testis, but also found in the brain. Expressed in prostate and ovarian cancer. Plays a crucial role in sperm migration and sperm–egg interaction.

Information has been gathered from UniProt (www.uniprot.org) and MEROPS databases (https://www.ebi.ac.uk/merops/) or from cited references.

a As deposited in the UniProt database.

b Accession numbers according to the Enzyme Commission (EC) and MEROPS databases.

c Refers to the full-length protein; residues Met1-Gly29 form the signal peptide and are removed, as well as the Ala30-Gly32 activation peptide. The C-terminal residues Pro323-His343 are reported to be removed during maturation.

d According to some authors, the protein is GPI-anchored instead.

e Refers to the full-length protein; the signal peptide, Met1-Arg19 is removed. The C-terminal propeptide, Gly289-Val314, is also cleaved during maturation.

f Refers to the full-length protein; the signal peptide, Met1-Gly19 is removed. The C-terminal propeptide, Thr300-Pro318, is also cleaved during maturation.

g Refers to the full-length protein; the signal peptide, Met1-Leu18, is removed, and the C-terminal residues Gly326-Tyr352 are also removed during maturation.

h Controversial. According to some authors, the protein is GPI-anchored instead.

Most MASPs possess basal activity in their zymogenic, single-chain forms and could generate full proteolytic activity after clustering on the cell membrane, which triggers autoactivation in *trans*. Prostasin, which does not autoactivate (20), has been shown to form an evolutionary conserved proteolytic cascade with matriptase essential for epithelial development (see *e.g.*, refs. (21, 22)). Further, matriptase is also activated by androgen-induced TMPRSS2 (23). These and other MASPs are predicted to function as initiators of cascades of trans- and coactivating proteases (16), which signal through important physiological substrates such as the G-protein-coupled receptor, protease-activated receptor 2 (PAR2), the epithelial sodium channel (ENaC), several matrix metalloproteinases (MMPs), and prohepatocyte growth factor (pro-HGF). This is in addition to only poorly understood cross talk between MASPs and the blood coagulation and fibrinolytic cascades, including the activation of urokinase-type plasminogen activator (uPA) (see *e.g.*, refs. (24, 25) and below).

### Colocalization of ACE2 and priming serine proteinases is essential for SARS-CoV-2 infection

ACE2 and TMPRSS2 colocalize on the surface of certain cell types and act, *de facto*, as an entry platform for SARS-CoV-1 and -2 (6). *ACE2* and *TMPRSS2* are coexpressed at particularly high levels in ciliated and secretory cells of the nasal cavity, which probably function as portals for initial infection by SARS-CoV-2 (26). The two genes and *FURIN* are primarily coexpressed in the lungs in a subset of bronchial cells differentiating from secretory to ciliated identity (27), in line with the more frequent and potentially life-threatening complication of COVID-19, pneumonia. Finally, high *ACE2* expression levels in differentiated enterocytes, the intestinal absorptive cells, might also explain the fact that the intestine is another important viral target (28).

However, it has now become evident that SARS-CoV-2 is capable of infecting not only cells of the respiratory and intestinal tracts but several other human organs as well, and COVID-19 is currently considered a systemic disease, for which the term “viral sepsis” has been recently coined (29). In this regard, it is noteworthy that *ACE2* and *TMPRSS2* are not coexpressed in the majority of tested cells (ref. (26); see also Fig. 4*A*). Such results suggest that other MASPs might either potentiate priming of the S protein in ACE2^+^/TMPRSS2^+^ cells (for instance, by simultaneously targeting two or all three S2’ sites in a trimeric S protein, thus exposing multiple fusion peptides at once) or even replace TMPRSS2 altogether in cells of organs/tissues that do not express significant levels of this serine proteinase.

### Noncatalytic domains do not influence the position of MASP active sites relative to the cell membrane

At first sight, it could be thought that members of the MASP subfamily with longer, more complex noncatalytic chains than TMPRSS2 would be less likely to function as activators of SARS-CoV-2 spike protein or to do so less efficiently (compare Fig. 2). However, several lines of evidence indicate that the actual MASP domain architecture is essentially irrelevant in this regard.

First, it is striking that diverse MASPs activate transmembrane or membrane-proximal substrates by cleaving the same peptide bond. Although matriptase is considered the most potent PAR2 activator (30, 31), activation of the GPCR by TMPRSS2, TMPRSS11D, and testisin (32, 33, 34, 35) as well as more poorly by hepsin (36) has also been reported (recently reviewed in ref. (37)). Further, TMPRSS3, TMPRSS4, matriptase, and prostasin have all been reported as ENaC activators (38, 39, 40, 41, 42). Several MASPs cleave and activate pro-uPA (24, 32, 43, 44, 45, 46), presumably bound to its cell surface receptor, uPAR, which is additionally targeted by TMPRSS11D (47). Both hepsin and matriptase have been identified as efficient, physiologically relevant activators of pro-HGF (43, 48, 49, 50) and of the matrix metalloproteinases, proMMP-1 and -3 (36, 51, 52). Finally, TMPRSS13 has also been reported as a pro-HGF activator (53).

On the other hand, regardless of the architecture of their noncatalytic chains, all well-characterized MASPs are inhibited by either one or both transmembrane inhibitors, hepatocyte growth factor activator inhibitor-1/serine proteinase inhibitor, Kunitz type 1 (HAI-1/SPINT1) or the related HAI-2/SPINT2 (see Fig. 4*B* for their domain organization). Kinetic analysis has been reported so far for hepsin inhibition by the first Kunitz domain of HAI-1 (54) as well as for the matriptase-HAI-1 pair (55, 56). In addition, HAI-1 and/or HAI-2 have been identified as physiologically relevant inhibitors of hepsin (50), TMPRSS2 (57, 58, 59), TMPRSS3 (57), TMPRSS4 (57, 60), matriptase-2 (61, 62), HATL1/TMPRSS11A (57), HAT/TMPRSS11D (63), DESC1/TMPRSS11E (64), TMPRSS13 (53, 57, 65), matriptase (58, 60, 66, 67, 68, 69, 70), enteropeptidase/TMPRSS15 (57), and prostasin (68, 71, 72, 73). In fact, HAI-2 has been recently proposed as a broad-spectrum antiviral agent (58).

The relative positions of SRCR and serine proteinase modules in the crystal structure of hepsin strongly suggest that the catalytic domain of the proteinase lies essentially flat against the plasma membrane (ref. (74); see also Fig. 3*B*). In the light of the shared substrate and inhibitor profiles of several MASPs discussed above, a similar localization should be expected for the catalytic domains of all other related, membrane-bound proteinases. Thus, the combined structural and functional evidence strongly suggests that the active sites of all MASPs would be located close to the cell membrane. An important consequence for SARS-CoV-2 activation is that, upon cleavage of the Arg815-Ser816 bond in the S protein, the exposed viral fusion peptide would be able to rapidly interact with the host-cell phospholipid membrane, thus allowing escape from immune surveillance.

Three major features would facilitate MASP access to the S2’ sites of an ACE2-bound, trimeric viral S protein: (1) the fluidity of phospholipid membranes, which allows lateral displacements of the viral protein and of its cellular receptors, (2) the high degree of structural plasticity of the spike protein, exemplified by the transition between “up” (ACE2 accessible) and “down” (ACE2 inaccessible) conformations, and finally (3) the flexibility provided to ACE2 and MASPs by some of their interdomain linkers, which would support both large rotations and displacements of protein modules relative to each other and to the cell membrane. The combined result would be the rapid (re)positioning of the S2’ sites of the spike protein in the catalytic cleft of the activating proteinase, in a conformation compatible with proteolysis of the Arg815-Ser816 bond.

### The catalytic domains of most MASPs could efficiently cleave the Arg815-Ser816 peptide bond in SARS-CoV-2 spike protein

Another issue arises when assessing the capability of MASPs other than TMPRSS2 to activate viral S proteins: could unique features of this serine proteinase favor recognition and proteolysis of the Arg815-Ser816 peptide bond? Despite highly divergent domain architectures (Fig. 2), all MASPs share well-conserved, trypsin-like catalytic domains. (Mechanism and specificity of serine proteases are reviewed in ref. (75)). Crystal structures have been reported for the catalytic domains of hepsin (48, 74, 76, 77) (see also Fig. 3, *B*–*C*), DESC1 (78), matriptase (79, 80, 81, 82, 83, 84, 85, 86, 87, 88), enterokinase (89), and prostasin (90, 91, 92), either free (unliganded) or, more commonly, bound to natural or synthetic inhibitors. This wealth of structural information allows generation of high-quality 3D models for the remaining members of the subfamily, including TMPRSS2.

A detailed analysis of the substrate and inhibitor specificity of all MASPs in the light of these structures/models has important implications for the mechanism of viral S protein cleavage. In particular, it can be predicted that most MASPs could target the Arg815(P_1_)-Ser816(P_1_’) bond of SARS-CoV-2 spike protein with similar efficiency as the canonical activator, TMPRSS2.† Prominent exceptions would be enterokinase and TMPRSS11B, which appear to have quite restricted substrate specificities: acidic residues at the P_4_–P_2_ positions and side-chain-less glycines at P_1_’ and P_2_’, respectively.

Thus, furin-mediated cleavage at the S1/S2 site would enhance infectivity of SARS-CoV-2 because S protein monomers would not need to be repositioned in the active site cleft of the activating MASP, but most importantly so because several MASPs would be able to attack the Arg815-Ser816 bond at a similar rate as TMPRSS2, which would not be the case if they had to target the S1/S2 site first. (Because bulky and basic arginine residues are present at positions P_3_ and P_4_ of this site (Fig. 1*A*), the Arg685-Ser686 peptide bond is much less likely to be efficiently targeted by multiple MASPs. Matriptase, for instance, prefers substrates with basic Arg/Lys residues at positions P_3_ or P_4_, but not both, and also TMPRSS9 has been shown to be a poor activator of substrates with polybasic sequences). Of note, the presence of a furin-cleavage site in the influenza fusion protein, hemagglutinin, sets apart low from high pathogenicity avian influenza viruses (93).

### Several MASPs are able to activate viral fusion machineries

In addition to TMPRSS2, other MASPs have been reported as activators of diverse viral fusion proteins, at least *in vitro*.‡ It was recently shown that TMPRSS2 and TMPRSS4 synergize to promote infection of small intestinal enterocytes by SARS-CoV-2 (94). TMPRSS13 had been previously shown to prime both SARS- and MERS-CoV S proteins (95), and a recent investigation suggests that it is even a more efficient activator of SARS-CoV-2 spike protein than TMPRSS2 (96). These authors also demonstrate significant priming potential of three members of the HAT/DESC subgroup of membrane serine proteinases, TMPRSS11D, TMPRSS11E, and TMPRSS11F, but only limited activity for TMPRSS11A and TMPRSS11B. By contrast, another recently presented work suggests that TMPRSS11A might process SARS-CoV-2 spike protein even more efficiently than TMPRSS2, at least *in vitro* (97). This is in line with previous reports on the activator activity of these MASPs: TMPRSS11A had been shown to activate MERS spike protein as well as hemagglutinin (64), TMPRSS11D primes SARS-CoV S protein for membrane fusion and also cleaves ACE2 (3, 98), while TMPRSS11E had been reported as an activator of SARS and MERS coronaviruses (95).

In addition to these reports on the activation of SARS-CoV-1/-2 and MERS-CoV by multiple MASPs, a large body of experimental evidence indicates that hepsin, TMPRSS4, TMPRSS11D, TMPRSS12, TMPRSS13, matriptase, and prostasin activate certain influenza strains among other respiratory viruses, although significantly less efficiently than TMPRSS2 in most cases (99, 100, 101, 102, 103, 104, 105, 106, 107, 108, 109, 110). Considering the experimentally verified involvement of certain MASPs in SARS-CoV-1/-2 priming in model systems, activation of the fusion process in other viruses, expression levels, and similarity of their catalytic machineries to that of TMPRSS2, a putative ranking of MASPs expected ability to prime SARS-CoV-2 spike protein can be postulated: TMPRSS4, TMPRSS13 > TMPRSS11D, 11E, 11F > TMPRSS11A > matriptase/prostasin > matriptase-2, hepsin, TMPRSS5 > testisin, matriptase-3, corin > TMPRSS9, TMPRSS12, PRSS41 > PRSS55, TMPRSS3 > TMPRSS1B > enterokinase.

### TMPRSS11B is unlikely to be an efficient activator of SARS-CoV-2 S protein but might be involved in an alternative pathway of viral cell entry

Analysis of MASP active site machineries suggests that only side-chain-less glycine residues would be well tolerated at the positions following a basic Arg/Lys-P_1_ residue in TMPRSS11B substrates. The only *bona fide* substrate of this MASP reported to date is the transmembrane protein, basigin (CD147/EMMPRIN) (111). The presence of a K↓GG site suggests that cleavage after Lys57 in the common basigin isoform would shed (“solubilize”) the receptor from the cell surface, as reported by Updegraff and coworkers.

Basigin has been proposed as an alternative receptor for SARS-CoV-2 S protein (112), although other authors have failed to detect interactions between the viral protein and human basigin (113). By contrast to ACE2, basigin is ubiquitously expressed at high levels, most notably in the heart. Of note, the receptor could also be targeted by TMPRSS11D (114), which reminds of the reported cleavage of ACE2 by TMPRSS2 and other MASPs. Along these lines, basigin had been previously identified as a receptor essential for erythrocyte invasion by the malaria parasite, *Plasmodium falciparum* (115), as well as for vascular colonization by *Neisseria meningitidis* (116), and has also been linked to cytomegalovirus infection of both endothelial and epithelial cells (117). Thus, the pathological relevance of a cell entry pathway involving basigin—and perhaps also its closest relatives within the immunoglobulin superfamily, neuroplastin and embigin—deserves careful examination, as emergence of a second high-affinity receptor for the spike protein in SARS-CoV-2 mutants might have catastrophic consequences.

### “Proteolytic surveillance”: endogenous MASP inhibitors are likely to regulate SARS-CoV-2 infection *in vivo*

The physiological activities of all MASPs are tightly controlled by endogenous proteinaceous inhibitors that belong to either the Kunitz or the serpin families (I1 and I4 in the MEROPS classification, respectively). The transmembrane members of the Kunitz family, HAI-1/SPINT1 and HAI-2/SPINT2, have been experimentally shown to inhibit the catalytic activity of every MASP tested so far. (See above and Fig. 4*B* for a schematic representation of HAI-1/-2 domain organization). Indeed, as expected from the high degree of sequence conservation at and around the active site of these proteinases, current structural information supports formation of stable MASP·HAI-1/HAI-2 complexes (80, 118). Overall, the two Kunitz inhibitors show a complementary pattern of expression in human organs/tissues, which would allow inhibition of MASP activity throughout the body, with the notable exception of the heart (Fig. 4, *C*–*D*). Thus, HAI-1 and HAI-2, as master regulators of all MASPs, would play an important role in SARS-CoV-2 propagation by regulating the availability of serine proteinase active sites on host cell membranes. Indeed, it has been recently shown that HAI-2 reduces cleavage/activation and growth of several influenza viruses by inhibiting TMPRSS2 or matriptase (58).

On the other hand, recently published results demonstrate that TMPRSS2 is targeted by the serpin, α1-antitrypsin (AAT/SERPINA1), which inhibits SARS-CoV-2 infection *in vitro* (119, 120). Notably, other reports show that AAT inhibits protease-mediated cell entry by SARS-CoV-2 at concentrations much lower than those found in serum and bronchoalveolar tissues, which suggests that the serpin is a physiologically relevant inhibitor of viral infection (121). AAT, however, is more specific for elastase-like serine proteinases, as indicated by the presence of a hydrophobic methionine as P_1_ residue. Noteworthy, several additional, trypsin-like MASPs (hepsin, all three matriptases, and DEC1) are potently inhibited *in vitro* by the most important circulating serpin, antithrombin (AT/SERPINC1) (122, 123, 124). A similar inhibitory capacity has been reported for other abundant serpins with broad specificities such as plasminogen activator inhibitor 1 (PAI-1/SERPINE1), α2-antiplasmin (α2-AP/SERPINF2), and protein C inhibitor (PCI/SERPINA5). Prostasin activity is regulated *in vivo* by the closest phylogenetic relative of PAI-1, protease nexin-1/SERPINE2 (125), which also potently inhibits matriptase, at least *in vitro* (126). Finally, the ubiquitously expressed SERPINB12 has been reported as a slow-binding inhibitor of hepsin (127). AT and other serpins interact with proteoglycans and glycosaminoglycans present in large amounts on cell membranes and in the extracellular matrix. This interaction allows serpin trapping near the cell membrane and therefore increases the probability of productive complex formation with active MASPs.

Similar to the transmembrane Kunitz-type inhibitors, the high degree of conservation of residues that shape the active-site clefts of these serine proteinases strongly suggests that major circulating serpins would also target most, if not all, members of the subfamily. Altogether, the fusion activation potential of TMPRSS2 and other MASPs *in vivo* would be critically codetermined by the concentrations of cognate inhibitors of the Kunitz and serpin classes and their relative affinities for the priming proteinase. It is likely that imbalance in the expression levels of MASPs, on the one side, and HAI-1/-2 and serpins, on the other, would determine the likelihood of SARS-CoV-2 infection of a given cell and thus ultimately of COVID-19 progression, similar to the well-documented situation in cancer (128, 129). I propose the term “proteolytic surveillance” to refer to this up to now unappreciated level of control of viral infection.

### Signaling, endocytosis, and shedding

I finally mention two only poorly understood features of MASPs, but that potentially impact the life cycle of these proteinases, and therefore their capacity to prime S proteins and contribute to viral infection. It is noteworthy that the cytoplasmic domains of several members of the subfamily possess consensus phosphorylation sites for protein kinase C and/or casein kinase II (reviewed in ref. (15)). This observation suggests that phosphorylation and perhaps also other posttranslational modifications of the cytoplasmic tails of MASPs could promote endocytosis. This is in addition to the presence of specific, short linear peptide motifs in the cytoplasmic domains of MASPs important for proteinase trafficking, as recently reported for matriptase (130).

The role of the endocytic pathway in the replication process of coronaviruses, and in particular of SARS-CoV-1/-2, remains disputed, with strong evidence indicating that major autophagy factors of the host cell are not required for viral infection (reviewed in ref. (112)). It is currently unknown whether engagement of ACE2 and TMPRSS2 or other MASPs by viral proteins elicits intracellular signaling pathways that might enhance cellular uptake of virions. Overall, it is believed that the MASP-mediated proteolytic cleavage and concomitant direct cell–cell fusion (the so-called “early” pathway) are the pathologically relevant events in SARS-CoV-1 infection of respiratory epithelia *in vivo*, while endocytic trafficking (the “late” pathway) plays a secondary role in this regard (131). Similar considerations would likely apply for SARS-CoV-2 and are probably at the heart of the poor results of autophagy inhibitors, chloroquine and its derivative, hydroxychloroquine, both for prophylaxis and treatment of COVID-19.

Another issue that deserves consideration is the frequent detection of soluble MASP forms (reviewed in ref. (15)). Although alternative splicing could explain the presence of some circulating variants of these proteinases, active shedding from the cell membrane upon proteolytic cleavage seems to be the predominant mechanism. At least in the cases of hepsin, TMPRSS2, and matriptase, the same proteinases are responsible for ectodomain shedding, following cleavage in *trans* (132). However, the identity of proteinases that shed other MASPs has not been established; matrix metalloproteinases (ADAMs and MT-MMPs) are also likely to play a role in this regard, as demonstrated for HAI-1. These “sheddases” might represent yet another control element of viral uptake (*e.g.*, the higher the sheddase activity, the lower the density of MASPs on cell membranes, and thus the lower the probability that a given cell would be infected).

## Implications for the cell tropism of SARS-CoV-2 and clinical manifestations of COVID-19

The presence of several MASPs capable of priming the spike proteins of SARS-CoV-2 virions, in synergy with or in place of TMPRSS2, offers straightforward explanations for clinical data on COVID-19 that remain unresolved to date (Table 2). Noteworthy, some family members are exclusively or predominantly expressed in male reproductive organs, alone four of them in the testis (TMPRSS12, PRSS21/testisin, PRSS41/TESSP-1 and PRSS55/T-SP1; Figs. 3*A* and 4*A*). Along these lines, ACE2 is expressed at relatively high levels in the testis, only second to the small intestine (Fig. 4*A*). These features, together with the upregulation of TMPRSS2 expression by androgens (23, 133), might explain the more severe complications of COVID-19 in male compared with age-matched female patients, including a much higher death rate (2.8 *versus* 1.7%; see *e.g.*, refs. (134, 135)). The relative contribution of MASPs other than TMPRSS2 to this sex bias is highlighted by the fact that the outcome of influenza infection is generally worse for women instead (136). (Of note, almost all MASPs reported to activate specific influenza strains—hepsin, TMPRSS4, TMPRSS13, and in particular, TMPRSS11D; see above—are predominantly expressed in the female, rather than in the male reproductive organs. Even the matriptase–prostasin pair is ubiquitously expressed and at similar levels in both male and female organs).

**Table 2:** Hypothesized correlations between expression patterns of MASPs other than TMPRSS2 and pathological observations in COVID-19 patients

Putative priming MASP	Relevant expression organ/tissue	Likely relevance for SARS-CoV-2 infection and COVID-19 pathology
TMPRSS12, PRSS21 (testisin), PRSS41 (TESSP-1), and PRSS55 (T-SP1)^a^ + TMPRSS6 (matriptase-2) + TMPRSS7 (matriptase-3)	Male reproductive organs (prostate, testis)	Possible viral reservoir. More severe complications of COVID-19 in male patients, leading to significantly higher mortality rates.
Airway trypsin-like proteases TMPRSS11A (HATL1), TMPRSS11D (HAT), TMPRSS11E (DESC1), and TMPRSS11F (HATL4) + TMPRSS4 + TMPRSS7 (matriptase-3) + TMPRSS14 (matriptase) + PRSS8 (prostasin)	At highest levels in the esophagus and minor salivary gland, but also expressed in the bronchi, trachea, and lungs	Alternative entry portals for SARS-CoV-2. Might contribute to and/or worsen lung infection/pneumonia.
TMPRSS5 (spinesin) + TMPRSS7 (matriptase-3)	Brain/tibial nerve	Might explain the neurological complications reported for some patients, as well as impaired motor functions.
TMPRSS6 (matriptase-2) + TMPRSS1 (hepsin)	Liver	Might contribute to liver damage.
TMPRSS10 (corin)	Cardiomyocytes	Might be responsible for heart damage. Might indirectly contribute to thrombotic complications through dysregulation of blood pressure.
TMPRSS1 (hepsin) + TMPRSS4 + TMPRSS14 (matriptase) + PRSS8 (prostasin)	Kidney	Might contribute to acute kidney injury (AKI).
TMPRSS14 (matriptase) + PRSS8 (prostasin)	Ubiquitously expressed in epithelial cells.	Involved in u-PA activation. Might be linked to thrombotic complications. Might also be linked to intestinal infection.

a Note that both PRSS41 and PRSS55 are termed “testis serine protease 1”.

These observations raise the hypothesis that male reproductive organs could be infected with SARS-CoV-2 and perhaps serve as viral reservoirs. Indeed, the testis is one of the organs in which more interactions between viral and host proteins were detected in a systematic study of the viral interactome, only second to the lungs (137). Of note, orchitis is a SARS complication, and the testes of patients who died of SARS-CoV-1 infection displayed widespread germ cell destruction with few or no spermatozoa (138). Persistent infection of this immune-privileged site by other unrelated, single-stranded RNA viruses has also been reported. Sertoli cells serve as reservoir for the Zika virus (139), and the testes are the preferred replication site of this flavivirus (140). Long-term testicular persistence of Ebola and Marburg viruses has been well documented and is responsible for sexual transmission of these filoviruses (141, 142).

The presence of multiple priming MASPs exposed on different human organs might account for the establishment of putative SARS-CoV-2 reservoirs. Identification of these reservoirs is an urgent need in the light of the growing number of patients with persistent COVID-19 symptoms (chronic or long COVID), as well as first reports of placental infection with SARS-CoV-2 (143, 144, 145) and vertical (transplacental) transmission of the virus to the fetus (146). In addition to the testes, other immune-privileged organs such as the eyes and brain might serve as sanctuary for the coronavirus.

The preferential expression of specific MASPs in organs/tissues that are well-known targets of SARS-CoV-2 infection is also striking (Fig. 3*A*) and may have important pathological implications. Most notably, members of the HAT/DESC subgroup, TMPRSS11A and 11D to 11F, together with other MASPs expressed in the airway tract (TMPRSS4, 7, 13, and 14), could contribute to the severe pneumonia observed in an important subset of COVID-19 patients. Further, engagement of corin/TMPRSS10 by SARS-CoV-2 in cardiomyocytes may explain the frequent myocardial damage reported in COVID-19 patients (147, 148). Myocardial infection by SARS-CoV-2 might be facilitated by the extremely low expression levels of HAI-1 and HAI-2 in the heart (Fig. 4, *B*−*C*), which suggests that the proteolytic activity of corin would be less tightly controlled than other members of the subfamily. Indeed, first autopsy findings reveal relatively high viral RNA titers in the heart, liver, brain, kidneys, blood, and/or gastrointestinal tract of several patients who died of confirmed SARS-CoV-2 infection (149, 150, 151). Also along these lines, SARS-CoV-2 RNA was detected in the heart and brain of infected mice in a recently presented model of the disease (152). The situation is also reminiscent of the systemic character of the influenza (H5N1) infection in cats, in which the virus could be detected most often in the heart, brain, liver, and kidney, in addition to respiratory tissues (153).

The matriptase–prostasin axis might also play an important role as viral activator, as these proteinases have the broadest expression patterns of all MASPs and are essential for the maintenance of epithelial barrier integrity. Indeed, infection of epithelial cells by SARS-CoV-2 in severe COVID-19 patients has been recently reported (154). The matriptase–prostasin pair could in particular contributes to intestinal infection.

It is also noteworthy that several MASPs are directly involved in plasminogen activation and fibrinolysis *via* pro-uPA cleavage (see ref. (24) and above). Matriptase, in addition, is engaged by blood coagulation factors upon activation of the tissue-factor-triggered clotting cascade (25). This unique location at the crossroads of epithelial signaling and the blood coagulation and fibrinolytic cascades could be particularly relevant in the light of reported thrombotic complications in COVID-19 patients (155). In addition to direct cardiomyocyte damage, these complications might be at least partly related to the “hijacking” of matriptase and other proteinases by SARS-CoV-2 and/or to their reduced expression/enhanced endocytosis, as part of negative feedback mechanisms. Finally, MASP engagement in other organs might explain a number of additional manifestations of COVID-19 reported so far, among them neurological complications including cognitive dysfunction (156) as well as acute kidney injury (151, 157).

## An integrated mechanism of SARS-CoV-2 spike protein cleavage and activation: an explanation for enhanced viral infectivity

**Figure 5:**
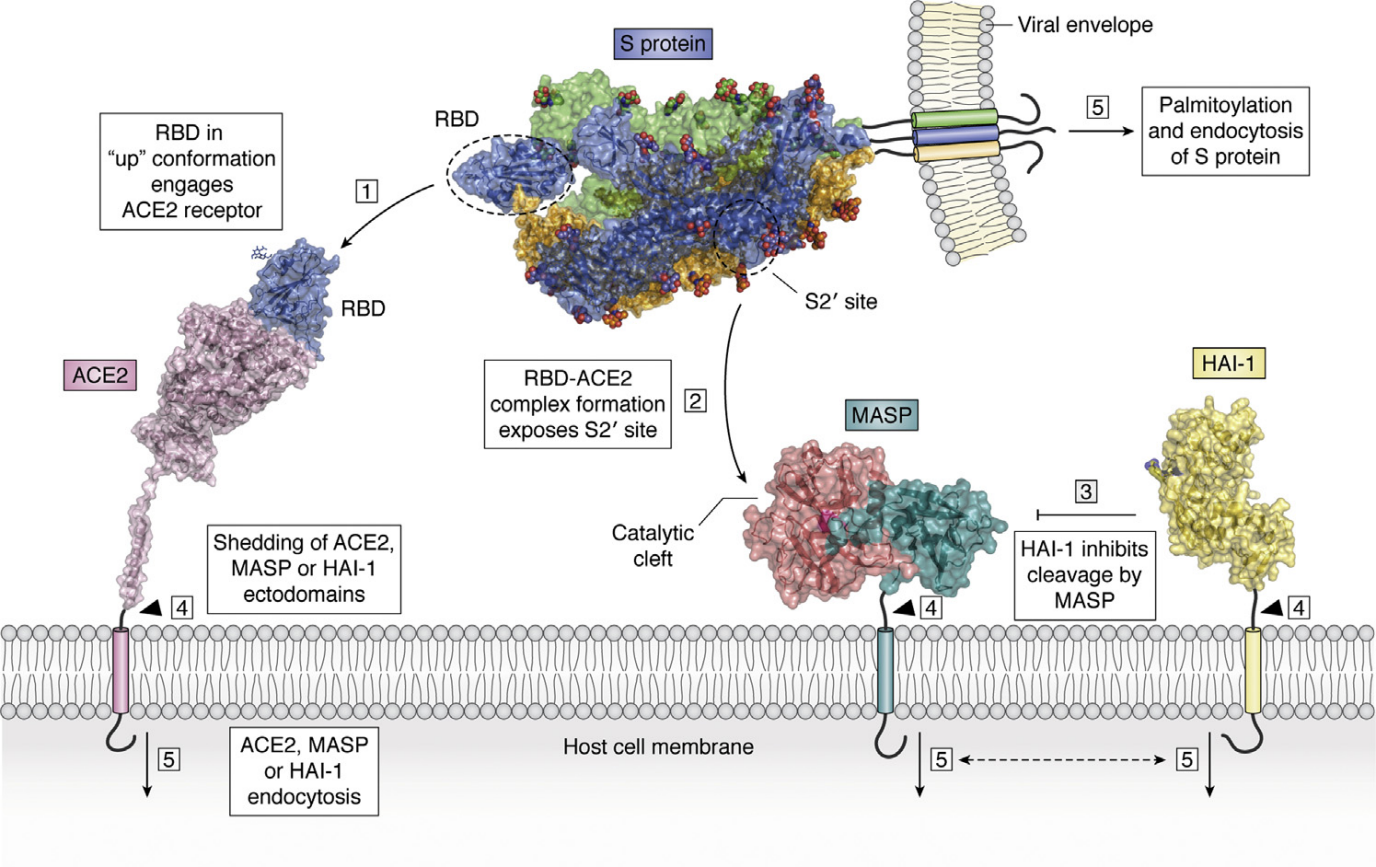
**Mechanism of S protein activation and viral–host cell membrane fusion.** The SARS-CoV-2 spike protein is found in the virions in a metastable prefusion state, in which the S1/S2 site has been cleaved by furin within the secretory pathway. (1) The RBD within the N-terminal S1 subunit of the S protein is exposed in the so-called “up” conformation and engages the host cell receptor, ACE2. (In the more stable “down” conformation, the RBD is not accessible to ACE2, which probably helps to evade immune surveillance, ref. (7)). A single ACE2 molecule is shown bound to the RBD of an S monomer, but two trimeric S proteins could simultaneously engage an ACE2 dimer *in vivo* (12). Also omitted for simplicity is the B^0^AT1 molecule that binds to the long C-terminal helix of ACE2 (after PDB 6M17). (2) RBD–ACE2 complex formation stabilizes a metastable conformation of the spike protein, stimulating “breathing” of the S2’ site and eventual exposure of the Arg815-Ser816 peptide bond to proteolytic cleavage by TMPRSS2 or another MASP. (3) MASP-mediated cleavage and activation of the S protein are controlled by endogenous inhibitors, most notably HAI-1 and/or HAI-2, but also by circulating serpins. Here, the HAI-1 ectodomain is shown, according to the reported crystal structure (PDB 5H7V; ref. (162)); the side chain of the P_1_ residue, Arg260, is highlighted. Other cellular processes that coregulate membrane fusion and viral uptake are (4) shedding of ACE2, MASPs and/or HAI-1/HAI-2 ectodomains, either by MASPs in *trans* or by membrane-bound metalloproteinases, and (5) endocytosis, which could be modulated by phosphorylation of cytosolic peptides in the human factors, or by palmitoylation of the Cys-rich endodomain of SARS-CoV-2. The scheme focuses on the “early”, endocytosis-independent pathway of virus cell entry. Endocytic trafficking (the “late” pathway) seems to play a minor role.

In summary, current structural and functional information suggests that several related, transmembrane trypsin-like serine proteinases decisively contribute to prime SARS-CoV-2 S protein either in synergy with or in place of TMPRSS2 and therefore promote infection of ACE2-coexpressing cells *in vivo*. A mechanism of viral protein activation that integrates current structural and functional information is schematically summarized in Figure 5.

The viral infection process could be viewed as a race, in which successful membrane fusion and infection depend on the balance between multiple competing protein–protein interactions. On the one hand, the viral spike protein requires simultaneous or highly synchronized binding to ACE2 and to the catalytic cleft of the priming MASP, in a conformation compatible with proteolysis of the S2’ cleavage site. Formation of these complexes and cleavage/activation of membrane fusion could be counteracted by circulating immunoglobulins directed against *e.g.*, the RBD in its exposed, “up” conformation, or against the fusion peptide exposed upon cleavage of the Arg815-Ser816 bond. In this regard, it is important to realize that S protein priming is followed by dramatic structural rearrangements, which lead to an unstable conformation of the S1 “crown” with all three RBDs up and its concomitant shedding. Thus, released S1 subunits might function as decoy antigens for circulating antibodies. This feature needs to be considered in the light of current efforts to develop vaccines based on immunization with the viral S protein or its RBD, as it might compromise the effectiveness of this approach. In addition to immune surveillance, the fusion machinery is under proteolytic surveillance by both proximal, transmembrane Kunitz inhibitors (HAI-1/HAI-2) andcirculating serpins. Finally, pericellular proteolysis of host proteins could either enhance virion uptake, as in the case of TMPRSS2-mediated cleavage of ACE2, or abort the whole membrane fusion process, upon MASP shedding.

An important element of the mechanism, revealed upon inspection of the 3D structures of SARS-CoV-2 S protein in its S1/S2-cleaved, prefusion conformation (13, 158), is the fact that the S2’ activation cleavage site is not accessible to proteolytic attack. Most notably, the side chain of the specificity-determining P_1_ residue, Arg815, occupies a cleft between two prominent α-helices comprising the N-terminal residues of the fusion peptide (Phe817-Asn824) and the following Asp867-Ile882 stretch, with its guanidinium group compensated by the carboxylates of neighboring acidic residues (Fig. 1, *C*−*D*). The side chain of this basic arginine residue stacks on the aromatic ring of Phe823, one of the critical residues for membrane fusion. The P_1_’ residue, Ser816, is even less accessible to bulk solvent, as it points toward the core of the subunit (Fig. 1*D*). In this manner, Arg815 and surrounding residues stabilize the prefusion conformation and avoid untimely exposure of the fusion machinery.

It follows that an important degree of flexibility (“breathing”) of the S2 subunit, in particular of residues surrounding the S2’ activation cleavage site, is required for formation of productive MASP-S protein complexes and proteolysis. Although the positions of Arg815 and neighboring residues are essentially conserved in the spike proteins of SARS-CoV-1 and MERS-CoV, there are also important sequence and structural differences, which might have a bearing on their relative infectivities (Fig. 1, *E*–*F*). In particular, the Arg815-pocket in SARS-CoV-1 and MERS-CoV is more acidic and overall better shaped to accommodate the Arg815 side chain. Therefore, I hypothesize that the S2’ activation cleavage site of SARS-CoV-2 spike protein would be more frequently exposed to activating proteinases than those of the related coronaviruses, which would contribute to its enhanced infectivity.

## Summary and perspectives

Unique adaptations of SARS-CoV-2 for human infection (*e.g.*, higher affinity of the S protein for human ACE2, its presence in a preactivated state in the virions following furin cleavage at the S1/S2 site, and its higher capacity for membrane fusion) are at the heart of the current COVID-19 pandemic. In addition, MASPs other than TMPRSS2 appear to prime SARS-CoV-2 S protein *in vivo*, which is likely to play an important role in determining the viral cell tropism as well as disease progression. Among the critical questions that remain unanswered are:

- Which MASPs serve as viral activating factors? The substrate and inhibitor profiles of these proteinases need to be carefully characterized, including structural investigations and rigorous quantum mechanical/molecular dynamics (QM/MD) simulations of their mechanism of S protein recognition and proteolysis.
- Which MASPs colocalize with human ACE2 in cells from different organs and tissues? Are there sex- and age-related differences that could contribute to explain current clinical observations, such as the higher mortality rate in male patients? Animal models might be useful to study the contribution of each MASP to SARS-CoV-2 infection of relevant cell types. (*e.g.*, by crossing transgenic mice overexpressing human ACE2 (152) with conditional knockouts of individual MASPs).
- Are there correlations between the levels of circulating forms of specific MASPs and their endogenous inhibitors (HAI-1/HAI-2 and serpins) and clinical parameters of hospitalized COVID-19 patients? In particular, do relative expression levels of these factors are related to differences in disease evolution such as mild *versus* severe respiratory distress and infection of organs/tissues other than the lungs? Are these levels predictive markers of disease evolution? (For instance, are higher relative concentrations of inhibitors predictors of a less virulent infection or even protect against infection?).
- Preliminary investigations provide direct evidence of SARS-CoV-2 infection in *e.g.*, the heart, liver, kidneys, and testes of COVID-19 patients (149, 150, 151). How frequently are these organs infected by the coronavirus, and does this frequency correlate with measured viral titers and the relative expression levels of specific MASPs? Which are the histopathological and ultrastructural consequences of viral infection in these organs?
- The correlation between COVID-19 and other comorbidities, in particular cancer, needs to be better understood. TMPRSS2 is an androgen-induced prostate cancer-specific marker, thus its overexpression is straightforwardly linked to a higher risk of infection in prostate cancer patients. Indeed, patients receiving androgen-depravation therapy showed a significantly lower risk of SARS-CoV-2 infection compared with those not treated with antiandrogens (159). Since most other MASPs are overexpressed in several cancer types (Table 1), also patients diagnosed with colorectal, skin, breast, or ovarian cancers are more likely to be infected and/or have more complications than sex- and age-matched individuals. Indeed, substantial rates of hospitalization and more severe outcomes have been reported in cancer patients diagnosed with COVID-19 (160). These patients deserve particularly close follow-up, and they should be advised to adopt the most restrictive measures suggested within their respective communities to avoid viral infection and transmission.

The answers to these questions would help diagnosis and treatment of the current COVID-19 pandemic but also future coronavirus diseases.

## Data availability

3D models of the catalytic domains of all MASPs not experimentally characterized to date have been deposited in the Protein Model DataBase (http://srv00.recas.ba.infn.it/PMDB/main.php). In all cases, residues are numbered according to the topology-based chymotrypsin numbering system, and coordinates have been superimposed on the hepsin crystal structure (PDB code 1P57) to facilitate comparisons.

## Conflict of interest

The authors declare that they have no conflicts of interest with the contents of this article.

## Footnotes

† According to the Schechter and Berger nomenclature, substrate residues are denoted as P_m_, …, P_1_, P_1_’, …, P_n_’, from N to C terminus; P_1_-P_1_’ is the scissile peptide bond to be cleaved by the proteinase. The cognate binding sites for these residues in the proteolytic enzyme are termed S_m_, …, S_1_, S_1_’, …, S_n_’; not to be confused with the viral S1/S2/S2’ subunits.

‡ Several trypsin-like serine proteinases cleave the SARS-CoV S proteins and thus function as entry cofactors *in vitro*, including trypsin itself, as well as the procoagulant factor, FXa, and the major fibrinolytic enzyme, plasmin. However, these proteinases are unlikely to act as pathologically relevant activators of S proteins *in vivo*.
